# Mutation Status of Pleckstrin Homology-Like Domain Family A Member 3 (PHLDA3) in the Radiation-Induced Insulinoma Cell Line RIN-5F

**DOI:** 10.7759/cureus.107903

**Published:** 2026-04-28

**Authors:** Takayoshi Kiba

**Affiliations:** 1 Department of Medical Technology, Faculty of Life Science, Okayama University of Science, Okayama, JPN

**Keywords:** insulinoma, mutation status, neuroendocrine tumors, phlda3, rin-5f

## Abstract

Background

Pleckstrin homology-like domain family A member 3 (*PHLDA3*) has emerged as an important tumor suppressor gene, particularly in pancreatic neuroendocrine tumors (PanNETs), such as insulinomas. *PHLDA3* is also known to function as a p53-inducible tumor suppressor and plays a critical role in regulating Akt signaling and apoptosis. It is also recognized as a key component of a radiation-responsive gene signature, highlighting its potential as a biomarker for radiation exposure. Previous studies have shown that *PHLDA3* mRNA is upregulated in irradiated mouse hematopoietic cells by quantitative RT-PCR. The RIN-5F cell line, a rat islet β-cell line derived from a radiation-induced insulinoma, is widely used in diabetes and endocrinology research. However, *PHLDA3* mutations have not previously been investigated in radiation-induced insulinoma cell lines, even though ionizing radiation is known to cause genetic abnormalities.

Methodology

A putative p53-responsive element (p53RE) overlapping the transcription initiation site of the *PHLDA3* gene has been identified. Because rat *PHLDA3* is a single-exon gene, it lacks intronic regions. This study examined the DNA mutation status of the single exon and the putative p53RE of the *PHLDA3* gene in the RIN-5F insulinoma cell line to determine whether radiation exposure induces mutations in *PHLDA3*.

Results

No mutations were detected in either the single exon or the putative p53RE of the *PHLDA3* gene in this cell line.

Conclusions

The study was designed as an initial exploratory analysis specifically focused on insulinoma, which has been reported to exhibit altered *PHLDA3* expression. Therefore, establishing whether genomic alterations exist in RIN-5F, one of the insulinoma cell lines, was a necessary first step. These findings suggest that radiation may promote the transformation of islet β-cells into insulinoma cells through mechanisms that do not involve direct mutations in the *PHLDA3* gene. This highlights the complexity of radiation-associated cellular transformation and suggests that factors other than *PHLDA3* mutational inactivation, such as epigenetic regulation or transcriptional changes, may contribute to islet β-cell oncogenesis.

## Introduction

Pleckstrin homology-like domain family A member 3 (*PHLDA3*) has emerged as an important tumor suppressor gene, particularly in pancreatic neuroendocrine tumors (PanNETs), such as insulinomas. Its tumor-suppressive function is primarily mediated through antagonism of the Akt signaling pathway, a central regulator of cell survival, proliferation, and metabolism [1]. Dysregulation of *PHLDA3* is not restricted to the pancreas; abnormalities in its expression or genomic status have also been reported in lung and rectal neuroendocrine tumors, suggesting that *PHLDA3* plays a conserved tumor-suppressive role across multiple neuroendocrine tissues [1]. Because Akt activation promotes cell growth and inhibits apoptosis, the ability of *PHLDA3* to block Akt signaling positions it as a critical molecular brake on tumorigenic processes [2].

*PHLDA3* is a direct transcriptional target of the tumor suppressor p53 and encodes a small protein containing a pleckstrin homology (PH) domain. This PH domain binds to membrane phosphoinositides, enabling *PHLDA3* to compete with Akt for access to PI(3,4,5)P₃ at the plasma membrane [2]. Through this competitive inhibition, *PHLDA3* acts as a dominant-negative regulator of the PI3K/Akt signaling cascade. The rat *PHLDA3* gene, located on chromosome 13, shares approximately 92% amino acid homology with its human counterpart (NCBI Gene ID: 363989). Rat *PHLDA3* encodes a 126-amino-acid protein with an estimated molecular weight of 15 kDa. Although the NCBI database annotates two exons for the rat *PHLDA3* gene, comparison of the cDNA sequence (NM_001012206.2) with the genomic DNA sequence (NC_086031.1) indicates that the coding region is contained within a single exon, suggesting a simpler gene structure than previously assumed. Also, because the rat *PHLDA3* gene is a single-exon gene, it does not have an intronic region.

Ionizing radiation is a potent inducer of DNA damage, generating a variety of lesions such as single-strand breaks, base modifications, and highly cytotoxic double-strand breaks [3]. When DNA damage exceeds the capacity for repair, p53 shifts its transcriptional program toward the activation of pro-apoptotic genes, including *PHLDA3*. Supporting this, studies in irradiated adult C57BL/6 mice have demonstrated significant upregulation of *PHLDA3* mRNA in hematopoietic cells 24 hours after exposure, as measured by quantitative reverse transcription-polymerase chain reaction (RT-PCR) [4]. Despite the established involvement of *PHLDA3* in radiation-responsive pathways, its mutational status has not previously been examined in radiation-induced insulinoma cell lines.

The RIN-5F cell line, derived from a radiation-induced rat insulinoma, is widely used in pharmacological, toxicological, and endocrine research due to its stable insulin-secreting phenotype. Given its origin, this cell line provides a valuable model for investigating the molecular consequences of radiation exposure in β-cells. Kawase T et al. also identified a putative p53-responsive element (p53RE) within the *PHLDA3* gene that overlaps the transcription initiation site, and this element is highly conserved across species [2]. In the present study, we examined the DNA sequence of the single exon and the p53RE of the *PHLDA3* gene in the RIN-5F cell line to determine whether radiation contributes to the development of *PHLDA3* mutations and whether such mutations may play a role in the transformation of islet β-cells into insulinoma cells. Clarifying whether *PHLDA3* is genetically altered in this model is essential for understanding its involvement in radiation-associated tumorigenesis and for refining our broader view of islet β-cell oncogenic pathways.

This study was designed as an initial exploratory analysis specifically focused on insulinoma, which has been reported to exhibit altered *PHLDA3* expression. Therefore, establishing whether genomic alterations exist in RIN-5F, one of the insulinoma cell lines, was a necessary first step. In the present study, it is hypothesized that RIN-5F cells harbor mutations either within the exon or within the p53-responsive element of *PHLDA3*, which could contribute to altered gene expression and impaired p53-mediated transcriptional activation.

## Materials and methods

Cell culture

RIN-5F cells (ATCC® CRL-2058™), originally derived from rat pancreatic islet cells, were obtained from the American Type Culture Collection (Manassas, VA, USA). Upon receipt, the cells were thawed and expanded according to ATCC guidelines to ensure stable growth characteristics prior to experimental use. The cell line was maintained in RPMI-1640 medium (Gibco, USA) supplemented with 10% heat-inactivated fetal bovine serum (FBS), 100 U/mL penicillin, and 100 µg/mL streptomycin, providing essential nutrients and preventing bacterial contamination.

Cells were cultured in T-75 flasks and monitored regularly under an inverted microscope to assess morphology and confluency. Cultures were maintained at 37 °C in a humidified incubator with 95% air and 5% CO₂, conditions that mimic the physiological environment and support optimal cell growth. The culture medium was replaced every 2-3 days, and cells were passaged at approximately 70-80% confluency using standard trypsinization procedures to prevent overgrowth and maintain consistent experimental conditions. Only cells within a defined passage range were used to minimize variability. RIN-5F cells were grown to full confluence prior to genomic DNA extraction to ensure sufficient material for reliable sequencing analysis.

Genomic DNA preparation

Genomic DNA was isolated from freshly collected cell samples derived from the cell line to ensure minimal degradation and optimal nucleic acid quality. Prior to extraction, the samples were briefly rinsed with phosphate-buffered saline (PBS) to remove residual culture medium and debris. Genomic DNA extraction was performed using the NucleoSpin® DNA RapidLyse Kit (Takara Bio Inc., Japan), following the manufacturer’s protocol to maintain consistency and reproducibility across experiments. This kit employs a rapid lysis procedure and silica-membrane purification, enabling efficient removal of proteins and other contaminants.

Cell samples were thoroughly homogenized to ensure complete cell disruption, and lysates were incubated under the recommended conditions to maximize DNA release. After binding to the purification column, samples were washed multiple times to remove residual salts and enzymatic inhibitors. The purified DNA was then eluted in nuclease-free buffer and stored at -20 °C until further analysis. Because RIN-5F represents a single, clonally derived cell line rather than a heterogeneous population of independent biological samples, genomic DNA was extracted on three separate occasions from independently cultured confluent cells. Each extraction was subjected to its own independent PCR amplification and sequencing analysis to ensure reproducibility and to confirm that the results were not influenced by batch-specific variation.

The concentration and purity of the extracted genomic DNA were assessed spectrophotometrically by measuring absorbance at 260 nm and 280 nm. The A260/A280 ratio was used to evaluate protein contamination, and only samples that met established quality criteria were used for downstream applications, including PCR amplification and sequencing.

PCR

A single exon and the p53-responsive element (p53RE) of the PHLDA3 gene were amplified by PCR to generate fragments suitable for downstream cloning and sequence verification. PCR amplification was performed using two high-fidelity DNA polymerases, PrimeSTAR® HS DNA Polymerase (Takara Bio Inc., Kusatsu, Japan) and KOD Plus Neo® (TOYOBO Inc., Osaka, Japan), to ensure accurate replication of the target sequence and minimize the introduction of nucleotide substitutions. These enzymes were selected for their proofreading activity and robust performance in amplifying GC-rich regions. To assess reproducibility, PCR amplification was conducted in triplicate with each of the two high-fidelity polymerases. The forward and reverse primers of the single exon and the p53RE of PHLDA3 are presented in Table 1. The primers were designed to include EcoRI and XhoI restriction sites at the 5′ and 3′ ends, respectively, enabling directional cloning of the PCR product into the pBlueScript II SK (-) vector (Stratagene, Santa Clara, CA, USA). Primer sequences were verified using NCBI Primer-BLAST to ensure specificity and to avoid off-target amplification.

**Table 1:** Sequences of the forward and reverse primers used for amplification and sequencing of the rat PHLDA3 gene. PHLDA3: Pleckstrin Homology‑Like Domain, Family A, Member 3.

Primer set	Primer	Sequence
Single exon	Forward (29 base pairs)	ATAGAATTCCGAGAAGCCGGCGTTGTAGA
Single exon	Reverse (29 base pairs)	ATACTCGAGGCTCTGGGACTTACCTGGCA
Putative p53-responsive element	Forward (29 base pairs)	ACGGAATTCAGGTTGATACCCACCTTGCC
Putative p53-responsive element	Reverse (29 base pairs)	ATACTCGAGCGGAAGTCGATTTGATGCGG

PCR reactions were prepared in sterile 0.2-mL microtubes on ice to prevent premature enzyme activity. Each 50-µL reaction mixture contained 5 µL of 10× PCR buffer, 5 µL of dNTPs (0.2 mM each), 3 µL of MgCl₂ (1.5 mM), 1 µL of each primer (10 µM), 1-2 µL of genomic DNA template (approximately 200 ng), 1 unit of DNA polymerase, and nuclease-free distilled water to bring the final volume to 50 µL. All reagents were mixed gently to avoid bubble formation, which can interfere with thermal cycling. Amplification was performed using an ASTEC thermocycler (Fukuoka, Japan). The cycling protocol consisted of an initial denaturation step, followed by 30 cycles of denaturation at 98 °C for 10 seconds, annealing at 55 °C for 10 seconds, and extension at 68 °C for 30 seconds per kilobase pair. A final extension step was included to ensure complete synthesis of all amplicons. Reaction products were analyzed by agarose gel electrophoresis to confirm the expected fragment size and assess amplification specificity.

PCR products that yielded a single, distinct band were purified using a standard gel extraction kit and subsequently subjected to Sanger sequencing. Sequencing was performed at Eurofins Genomics (Tokyo, Japan) using a 3730xl DNA Analyzer (Thermo Fisher Scientific, Tokyo, Japan). Because PCR was performed in triplicate, each of the three PCR products was sequenced independently to ensure reproducibility. PCR reactions also included a wild-type PHLDA3 template as a positive control and a no-template control to monitor potential contamination. Sequence chromatograms were inspected manually using Chromas version 2.66 (Technelysium Pty Ltd., South Brisbane, Australia) to verify the absence of PCR-induced mutations and to confirm the correct orientation of the amplified fragment. The absence of mutations was defined using the following criteria: (1) bidirectional Sanger sequencing produced identical sequences; (2) chromatograms showed no mixed or ambiguous peaks; (3) all bases were clearly resolved with a high signal-to-noise ratio; and (4) the obtained sequence matched the reference PHLDA3 sequence exactly.

Ethics and consent to participate

All experimental procedures involving recombinant DNA were conducted in strict accordance with the university’s Guidelines for Recombinant DNA Experiments. These guidelines outline institutional standards for biosafety, risk assessment, and the proper handling of genetically modified materials, and full compliance was maintained throughout the study. Prior to initiating any experimental work, the research plan underwent formal review to evaluate potential biosafety concerns and to ensure adherence to both national and institutional regulations.

The protocols used in this study were reviewed and approved by the university’s Safety Committee for Recombinant DNA Experiments (approval number 1714). This approval confirms that the experimental design, laboratory environment, and operational procedures met the required biosafety level and ethical standards. All experiments were conducted within designated containment facilities, and appropriate measures were implemented to minimize environmental release and ensure the safety of laboratory personnel. No human participants or animal subjects were involved in this study; therefore, additional ethical approval or informed consent was not required.

## Results

The rat *PHLDA3* gene, which consists of a single exon, lacks an intronic region. The single exon (378 base pairs) and the p53-responsive element (p53RE; 20 base pairs) of the *PHLDA3* gene were amplified and cloned by PCR using two high-fidelity DNA polymerases: PrimeSTAR® HS DNA Polymerase (Takara Bio Inc., Kusatsu, Japan) and KOD Plus Neo® (TOYOBO Inc., Osaka, Japan). To ensure amplification fidelity and reproducibility, PCR was performed in three independent replicates with each polymerase. These enzymes were selected for their strong proofreading activity and high accuracy, both of which are essential for minimizing PCR-induced mutations during amplification of coding regions. The use of two distinct polymerases also enabled cross-validation of amplification efficiency and fidelity, thereby increasing the reliability of the cloned DNA fragments.

**Figure 1:**
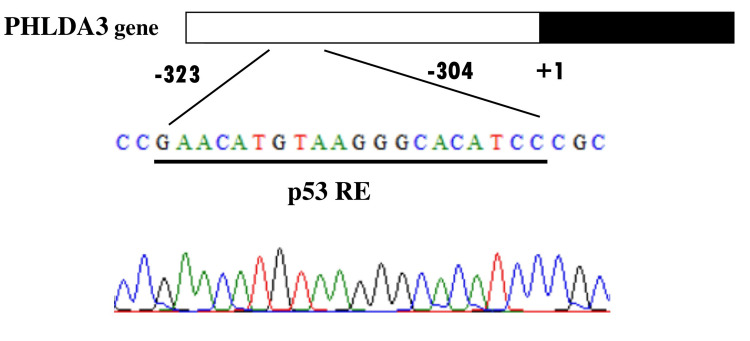
DNA sequencing analysis of the p53-responsive element (p53RE) within the PHLDA3 gene. The chromatograms show the nucleotide sequence of the p53RE region, confirming the absence of mutations in the RIN-5F cell line. PHLDA3: Pleckstrin homology-like domain family A member 3.

Genomic DNA was extracted from RIN-5F cells, which were confirmed to carry no mutations within the single exon or the p53RE of the *PHLDA3* gene (Figure 1). The three independent PCR products generated using PrimeSTAR® HS DNA Polymerase and KOD Plus Neo® were cloned separately. Amplification with both polymerases produced consistent and reproducible results, demonstrating that the exon sequence could be reliably amplified regardless of the enzyme used. These PCR products were subsequently subjected to downstream cloning and sequence verification to ensure that no nucleotide alterations were introduced during the amplification process.

## Discussion

The study was designed as an initial exploratory analysis specifically focused on insulinoma, which has been reported to exhibit altered *PHLDA3* expression. Therefore, establishing whether genomic alterations exist in RIN-5F, one of the insulinoma cell lines, was a necessary first step.

**Figure 2:**
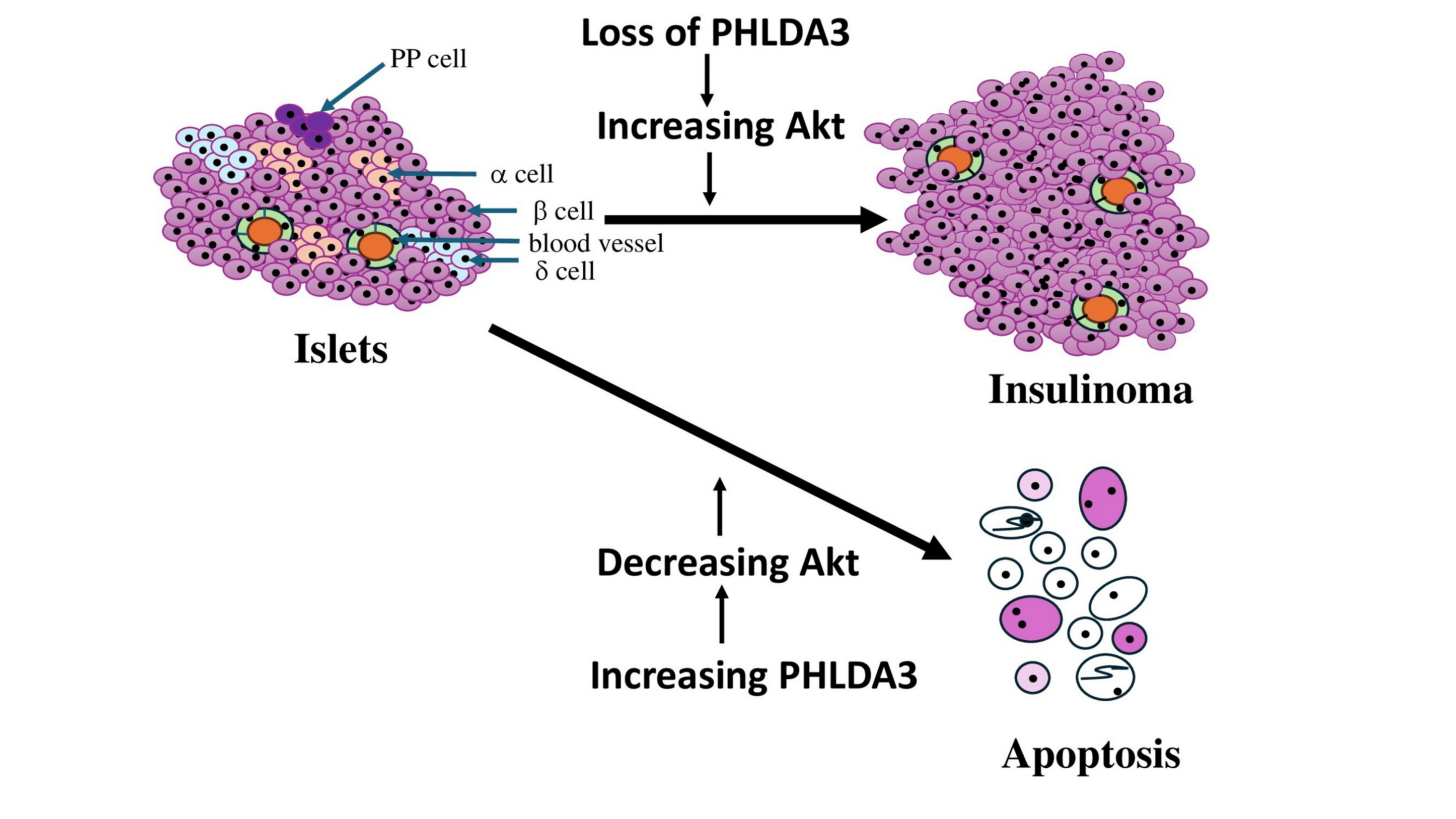
Schematic representation of the relationship between PHLDA3 expression and its effects on islet β-cells. The diagram illustrates how *PHLDA3* modulates AKT signaling and thereby influences β-cell survival and apoptosis. PHLDA3: Pleckstrin homology-like domain family A member 3.

Pancreatic regeneration, carcinogenesis, and apoptosis represent central themes in contemporary medical and molecular research, as these processes are closely linked to the development and progression of pancreatic diseases, including diabetes and PanNETs [5]. Among the molecular regulators implicated in these pathways, *PHLDA3* has gained increasing attention. Loss or reduced expression of *PHLDA3* is frequently observed in PNETs, particularly insulinomas, supporting its proposed role as a tumor suppressor in endocrine pancreatic tissue (Figure 2) [1]. Previous studies have demonstrated that *PHLDA3* plays a crucial role in regulating islet β-cell homeostasis. Investigations examining the relationship between *PHLDA3* deficiency, islet β-cell hyperplasia, and apoptosis resistance have identified heightened AKT activity as a key driver of β-cell proliferation, ultimately leading to hyperplasia [1]. The AKT pathway is well known for promoting cell survival by suppressing apoptotic signaling cascades. Consistent with this, *PHLDA3*-deficient mice exhibit marked resistance to apoptosis within their islet β-cells, underscoring the importance of *PHLDA3* as a negative regulator of AKT-mediated survival signaling [1]. The combined effects of enhanced proliferation and reduced apoptosis result in a pronounced expansion of β-cell mass, providing mechanistic insight into how *PHLDA3* loss may contribute to tumorigenesis (Figure 2).

In addition to its role in tumor biology, *PHLDA3* has been implicated in the maintenance of islet β-cell function under metabolic stress. Bensellam M et al. [6] reported that *PHLDA3* is essential for preserving β-cell viability in the context of type 1 and type 2 diabetes, conditions characterized by chronic exposure to inflammatory cytokines, oxidative stress, and endoplasmic reticulum stress. These findings underscore the broader physiological relevance of *PHLDA3* beyond oncogenesis, positioning it as a key regulator of β-cell resilience under pathological conditions.

The RIN-5F cell line, derived from a rat radiation-induced insulinoma, serves as a widely used model for studying islet β-cell biology, insulin secretion, and radiation-induced cellular responses [7]. Exposure to ionizing radiation generates a spectrum of DNA lesions, including single-strand breaks, base modifications, and the more severe double-strand breaks. These lesions activate a complex network of DNA damage response pathways involving DNA-dependent protein kinase, ataxia telangiectasia mutated (ATM), and ATM- and Rad3-related (ATR) kinases [3]. Although the core components of these pathways have been well characterized, many radiation-responsive signaling events remain incompletely understood, and emerging studies continue to reveal additional regulatory mechanisms [8-11].

Among the key regulators of the radiation response is the tumor suppressor protein p53, which orchestrates cell-cycle arrest, DNA repair, and apoptosis depending on the severity of the damage [12,13]. *PHLDA3* has recently been identified as part of a radiation-responsive gene signature, suggesting its potential utility as a biomarker for radiation exposure and cellular stress assessment. When DNA damage is moderate and repairable, cells may restore genomic integrity and resume proliferation. However, in cases of severe or irreparable damage, p53 shifts its transcriptional program toward the activation of pro-apoptotic genes such as *PHLDA3*, *BAX*, *PUMA*, and *NOXA* [14].

In the present study, we examined whether radiation exposure induces mutations in the *PHLDA3* gene that could contribute to islet β-cell transformation and insulinoma development. Genomic DNA sequencing of the RIN-5F cell line clone demonstrated no detectable mutations in the single exon or the p53RE of the *PHLDA3* gene, suggesting that radiation-induced tumorigenesis in this model does not arise from direct mutational inactivation of *PHLDA3*.

Limitations

Several limitations should be acknowledged when interpreting the findings of this study. First, the investigation focused exclusively on the RIN-5F cell line, a rat insulinoma-derived β-cell model generated through radiation exposure. Although this cell line is widely used in endocrinology and radiation biology research, it does not fully recapitulate the complexity of primary islet β-cells or human PanNETs. Species-specific differences in DNA repair capacity, p53 signaling, and *PHLDA3* regulation may limit the generalizability of these results to human physiology or disease.

Second, the study examined only the genomic DNA sequence of *PHLDA3* to determine whether radiation-induced mutations contribute to islet β-cell transformation. Although no mutations were detected within the single exon or the p53RE, this approach does not exclude the possibility of epigenetic alterations, such as promoter methylation, histone modifications, or changes in chromatin accessibility, that are known to influence *PHLDA3* expression. Additionally, post-transcriptional and post-translational regulatory mechanisms, including microRNA-mediated repression, protein stability, and subcellular localization, were not assessed and may play important roles in modulating *PHLDA3* function under radiation stress.

Third, the study did not evaluate the broader genomic landscape of the RIN-5F cell line. Radiation exposure typically induces mutations across multiple genes involved in DNA repair, apoptosis, and cell-cycle regulation. Consequently, it remains unclear whether alterations in other pathways, rather than *PHLDA3* itself, contribute to insulinoma development in this model. Future studies involving whole-genome sequencing or targeted analysis of additional p53-responsive genes are needed to improve understanding of radiation-induced tumorigenesis.

Finally, functional assays assessing *PHLDA3* protein expression, AKT pathway activity, or apoptosis induction were not performed. As a result, the study cannot determine whether *PHLDA3* signaling is functionally altered despite the absence of detectable genomic mutations. Incorporating molecular assays such as Western blotting, reporter-based transcriptional activity measurements, or phospho-AKT analysis would strengthen the mechanistic conclusions by directly evaluating the functional status of the *PHLDA3*-AKT axis.

## Conclusions

The findings of this study suggest that radiation may promote the transformation of islet β-cells into insulinoma cells through mechanisms that do not involve direct mutations in the single exon or the p53RE of the *PHLDA3* gene. These results highlight the complexity of radiation-associated cellular transformation and indicate that factors other than *PHLDA3* mutational inactivation, such as epigenetic regulation or transcriptional reprogramming, may contribute to islet β-cell oncogenesis.

This conclusion underscores the importance of examining broader genomic and signaling networks when investigating radiation-induced endocrine tumors. While *PHLDA3* remains a key mediator of apoptosis and a potential biomarker of radiation exposure, its intact genomic status in this model suggests that its role in tumor development may be more nuanced than previously assumed. Future studies incorporating functional assays, epigenetic profiling, and genome-wide analyses will be essential to fully elucidate the molecular events driving islet β-cell transformation following radiation exposure.
